# Therapeutic and Metagenomic Potential of the Biomolecular Therapies against Periodontitis and the Oral Microbiome: Current Evidence and Future Perspectives

**DOI:** 10.3390/ijms232213708

**Published:** 2022-11-08

**Authors:** Simona Santonocito, Salvatore Ferlito, Alessandro Polizzi, Vincenzo Ronsivalle, Rossana Sclafani, Alessandra Valletta, Antonino Lo Giudice, Raffaele Cavalcanti, Gianrico Spagnuolo, Gaetano Isola

**Affiliations:** 1 Department of General Surgery and Surgical-Medical Specialties, School of Dentistry, University of Catania, Via Sofia 78, 95125 Catania, Italy; 2 Department of Medical and Surgical Sciences and Advanced Technologies “GF Ingrassia”, University of Catania, 95123 Catania, Italy; 3 Department of Neurosciences, Reproductive and Odontostomatological Sciences, University of Naples “Federico II”, 80138 Napoli, Italy

**Keywords:** periodontitis, adjuvants, microbial, antimicrobials, biofilm, host response, genetics, therapy, immunomodulation, metagenomic

## Abstract

The principles of periodontal therapy are based on the control of microbial pathogens and host factors that contribute to biofilm dysbiosis, with the aim of modulating the progression of periodontitis and periodontal tissue destruction. It is currently known how differently each individual responds to periodontal treatment, depending on both the bacterial subtypes that make up the dysbiotic biofilm and interindividual variations in the host inflammatory response. This has allowed the current variety of approaches for the management of periodontitis to be updated by defining the goals of target strategies, which consist of reducing the periodontopathogenic microbial flora and/or modulating the host-mediated response. Therefore, this review aims to update the current variety of approaches for the management of periodontitis based on recent target therapies. Recently, encouraging results have been obtained from several studies exploring the effects of some targeted therapies in the medium- and long-term. Among the most promising target therapies analyzed and explored in this review include: cell-based periodontal regeneration, mediators against bone resorption, emdogain (EMD), platelet-rich plasma, and growth factors. The reviewed evidence supports the hypothesis that the therapeutic combination of epigenetic modifications of periodontal tissues, interacting with the dysbiotic biofilm, is a key step in significantly reducing the development and progression of disease in periodontal patients and improving the therapeutic response of periodontal patients. However, although studies indicate promising results, these need to be further expanded and studied to truly realize the benefits that targeted therapies could bring in the treatment of periodontitis.

## 1. Introduction

Periodontitis is an infectious disease characterized by inflammation of the supporting tissues of the tooth. If not properly treated, it can lead to the destruction of periodontal tissues and tooth loss in the long term [1].

The onset and successive progression of periodontitis represent an unbalanced immune reaction of the host against an organized dysbiotic biofilm. The relationship between immune surveillance and the host immune response induced by oral microbes may be variable between individuals. If local stimulation induces a mild host immune response, immunological surveillance and an appropriate immune response predominate. Conversely, if the pathogenicity of the local microbiota is elevated by the colonization of keystone pathogens that overactivate the host immune response, tissue destruction is initiated [2].

Among the main known periodontal pathogens are bacteria such as Actinobacillus actinomycetemcomitans and Porphyromonas gingivalis (*P. gingivalis*), which have multiple factors underlying the tissue damage seen during periodontitis, such as peptidoglycans, various integrins and outer membrane proteins, lipopolysaccharides, and superficial cell fimbriae of connective tissue degradation [3,4,5,6,7,8]. Once these pathogenic bacteria trigger immune and inflammatory processes, there is a contextual host response with the local and systemic release by leukocytes, fibroblasts or other derived cells of various inflammatory mediators, including metalloproteinases, cytokines, transglutaminases, prostaglandins, and proteolytic enzymes [9,10,11]. More specifically, proteases cause collagen degradation in periodontal tissues and, therefore, can induce further leukocyte infiltration [12]. Despite the production and release of various inflammatory mediators, in the active phase of periodontal disease, tissue destruction occurs mainly due to an imbalance between the level of matrix metalloproteinases and their endogenous inhibitors [13]. Subsequently, through the stimulation of certain pro-inflammatory cytokines, such as interleukin (IL)-1b, IL-6, tumor necrosis factor and (TNF)-α, inflammatory infiltrates from periodontal tissues initiate tissue and alveolar bone destruction [7,13,14,15,16,17,18,19,20]. During periodontitis, activated osteoclasts and inflammatory-related mediators can control the release of ligand-receptor factor kappa B (NF-κB), receptor activator of nuclear factor kappa-Β ligand (RANKL), and osteoprotegerin (OPG), which activates the osteoclast receptor and the relative alveolar bone destruction [21]. Furthermore, it has been shown that different types of cytokines and growth factors can regulate osteoclastogenesis, thus increasing the diversity, proliferation, duration, and action of osteoclasts [22] such as calcitriol, proteins of the parathyroid-hormone, prostaglandin-E (PGE) -2, IL-1b, IL-6, and IL-11 [23]. However, the activation of osteoclasts needs the stimulation of macrophage colonies and requires an interaction between the macrophage and monocyte ancestors and osteoblast lineage, bone marrow stromal units, or the presence of T and B lymphocytes. All these complexes express RANKL, a key mediator in osteoclast activation [24]. Therefore, it is essential that RANKL binds to the receptor activator of nuclear factor kappa-Β (RANK), expressed on the cell surface of osteoclasts, to initiate osteoclastogenesis. This connection allows the release and differentiation of bone cells by macrophage and monocyte precursors and the production of mature osteoclasts [25]. In this respect, osteoprotegerin, a receptor on bone marrow cells and osteoblasts, significantly inhibits the RANKL/RANK interaction [26,27,28]. In periodontal lesions of *A. actinomycetemcomitans*-infected, CD4+ T lymphocyte subtypes have been shown to induce increased expression of RANKL in the presence of certain periodontal pathogens [18]. This leads to activation of osteoclasts and bone loss, which ultimately increases the microbial load that propagates the destructive periodontal lesion [26].

Currently, the first-line therapy of periodontal disease is scaling and root planning (SRP) using ultrasonic instruments, which induce the breakdown and mechanical removal of bacterial biofilm, resulting in a reduction of the inflammatory process [27]. However, patients do not always respond positively to such treatment, and there may be multiple causes, such as sites with deep periodontal pockets and teeth with complex anatomy, where ultrasonic instruments fail to totally remove the biofilm, or an excessive host inflammatory response [28]. Mechanical control of biofilm can therefore be adjuvanted with specific antimicrobial agents, such as antibiotics (both topical and systemic) [28], antiseptic agents [29], and drugs that modulate the host inflammatory response [30]. Such adjuvant therapies, however, have multiple side effects when used for prolonged periods, such as antibiotic resistance, oral tissue discoloration, etc. [28].

In light of what has been described, inflammation induced by dysbiosis produces, through the mediation of dysfunction and tissue damage, the growth of selectively dysbiotic (inflammophilic) communities of bacteria, thus generating a self-sustaining feed-forward cycle that perpetuates disease. Based on these observations, research has postulated the possibility of developing target therapies based on modulation of the immune microenvironment and associated with reducing periodontopathogenic microbial flora [30]. The aim of such therapies is to halt the progression of periodontitis and alveolar bone loss through periodontal biofilm analysis or modulation of the host response. In this regard, several studies have attempted to describe the main bacterial species that cause tissue damage in periodontitis and what are the novel therapeutic effects of reducing bacterial and inflammatory mediators released during periodontitis with targeted therapies and the possible beneficial effects in periodontal regeneration [31]. However, many aspects still need to be investigated, such as the real long-term benefits and risks.

Therefore, the purpose of the present study is to update current knowledge on approaches for the management of periodontitis based on recent targeted therapies, in order to understand their possible benefits, limitations, and future prospects.

## 2. Materials and Methods

The review of current literature for conducting this narrative review was conducted by consulting the following search engines Medline, Scopus, Cochrane, and Embase. The search was conducted using the following keywords: “periodontitis,” “periodontitis and target therapy,” “periodontitis and new therapy,” “periodontitis and immunotherapy,” and “periodontitis and biofilms.” RCT studies, prospective cohort studies, case-control studies, case-series and systematic and narrative reviews were included, covering the time frame from 1998 to 2022

## 3. Target Therapies for Periodontitis

In recent decades, several highly innovative analytical methods have been developed to further analyze the composition and dynamics of microbial communities and their interaction with the host [32]. However, although metagenome-specific assembly algorithms [33] and methods for clustering genomes from metagenome data [34] have presented encouraging results, especially for functional analyses of these data, only through proper sequencing and analysis of DNA and RNA alteration mediators will it be possible to obtain early markers of disease risk.

During periodontitis, a dysbiotic, well-organized biofilm manages to resist the host’s defensive mechanisms and, thanks to a nutritionally favorable inflammatory environment, find the optimum conditions for further growth. In the future, there will be improvements in medical diagnostics, with highly precise and minimally invasive methods followed by appropriate therapeutic concepts [31]. With this concept in mind, at present, saliva and similar fluid mediators, such as gingival crevicular fluid, represent diagnostic materials with enormous potential to provide information on various microbial and inflammatory biomarkers closely related to periodontitis, thanks also to a refinement of these diagnostic techniques [35]. In this regard, salivary analyses aimed at identifying a combined therapeutic technique of exploring the genome of the oral biofilm at the individual level, using the individual’s own metagenomic codes, should allow the analysis of individual-specific microbiomes useful for identifying the state of health and the possible state of disease [36,37]. This new personalized molecular approach could, in the future, change the current diagnostic viewpoint regarding the prevention and treatment of periodontitis [38], allowing therapy to be personalized to the characteristics expressed by the patient.

## 4. Cell-Based Periodontal Regeneration

Recent discoveries about the regenerative capacity of the periodontium, its stem cells and their regenerative possibilities have allowed the introduction of an innovative cell-based therapeutic approach to improve the efficacy and quality of periodontal regeneration [39]. Regeneration of periodontal tissue requires restoration of alveolar bone height in relation to the cementoenamel junction; regeneration of gingival connective tissue destroyed by inflammation; formation of new extrinsic acellular cement, usually located in the occlusal half of the root, and cellular cement, covering the most apical portion of the root surface, on previously exposed root surfaces; and synthesis of a functionally oriented periodontal ligament (PDL) [40,41]. Unfortunately, no conventional treatment aimed at decontamination of the root surface alone to halt disease progression (including scaling and root planing, open debridement) or in combination with guided tissue regeneration (GTR) using different biomaterials (bone derivatives and bone substitutes), or autogenous block grafting can ever predictably regenerate lost periodontal tissues (bone–PDL–cement) or reconstruct normal structure and function. Currently, biomaterials used are distinguished into bioactive agents consisting of biological or synthetic agents with both antibacterial and osteogenic effects; guided tissue regeneration/guided bone regeneration (GTR/GBR) membranes consisting of natural or synthetic polymers, combined with antibacterial and osteogenic components, which act as cellular barriers to prevent invasion of epithelium and connective tissues, promoting alveolar bone regeneration; and tissue engineering scaffolds which are composed of natural or synthetic polymers, antibacterial components, proteins or cells, whose action is to maintain space, store growth factors, and support attachment and cell proliferation when grafted onto the bone defect [42]. It is therefore believed that periodontal therapy should not be limited exclusively to stopping the inflammatory process induced by the removal of biofilm but should be supplemented with the regeneration of periodontal tissues lost as a result of the disease [43]. It is currently known that the periodontium has a limited capacity for self-regeneration: following the removal of biofilm from the affected tooth roots, the periodontal lesion will be repaired by the epithelial cells of the gingiva. Several experimental and human clinical studies have observed that although clinically conventional regenerative periodontal therapy results in a clinical attachment gain, it does not achieve complete and functional periodontal regeneration [44]. Periodontal tissue engineering, which makes use of appropriate cellular resources, is still under investigation in the periodontal field. In periodontal tissue engineering, mesenchymal cells have been applied to simulate simultaneous regeneration of the various components of the attachment apparatus, i.e., the alveolar bone, cementum, and periodontal ligament [45]. In particular, periodontal cell regeneration has been performed according to various approaches and principles that have been reported in numerous excellent reviews [46,47]. In the study carried out on dogs by Nakahara et al., autologous implantation of periodontal ligament cells seeded on a collagen sponge scaffold was performed in a model of periodontal fenestration defect. These authors observed this method’s complete regeneration of alveolar bone and root cement [48]. Furthermore, it was believed that the populations of progenitor cells of the periodontal ligament exhibited some characteristics of stem cells.

In this regard, Xu et al., in an in vitro study carried out on a set of stem cells obtained from the periodontal ligament of humans, showed that these mesenchymal stromal cells had similar features to mesenchymal stem cells, including multipotency, and the replication and high capacity of the proliferation of stem cell markers STRO-1 and perivascular CD146 [49]. Sonoyama et al. have shown the prospect of reconstructing an entire dental and periodontal ligament by implanting block stem cells of the dental root together with hydroxyapatite/tricalcium phosphate, coated with gel foam containing mesenchymal periodontal stromal cells of the ligament, in minipig tooth cavities [50]. Moreover, Liu et al. demonstrated that autologous mesenchymal stromal cells originating from the periodontal ligament were able to promote the healing of experimental periodontitis in minipigs and act as markers of progenitor cell genes [51]. The periodontal ligament obtained by cell culture was also considered a further cellular source suitable for stimulating regeneration. It can differentiate into osteoblastic cells and express particular tissue-related periodontal genes [52].

In this regard, Yamamiya et al. evidenced that the periodontal ligament periosteal cells in culture combined with platelet-enriched plasma and induced hydroxyapatite determined better healing of human vertical periodontal defects [53]. In different animal models, it has been observed that mesenchymal stem cells originating from bone marrow can effectively regenerate both alveolar bone and periodontal tissues. In an experiment performed on beagle dogs, Kawaguchi et al. showed that autograft mesenchymal stem cells derived from bone marrow determined a complete periodontal regeneration in experimental class III furcation defects [54]. Furthermore, by immunohistochemical analysis, the same group detected that the transplanted mesenchymal stem cells one month after transplantation were able to survive and differentiate into periodontal tissue cells, with consequent improvement of the periodontal tissue regeneration process [55]. Yamada et al. have shown the possibility of successfully regenerating periodontal tissue using autologous mesenchymal stem cells derived from bone marrow combined with platelet-rich plasma [56]. For adult patients, mesenchymal cells can be present in a wide range of tissues, such as stem cells derived from dental pulp or adipose tissue [57,58]. Similarly, the periodontal ligament houses niches of stem cells important in maintaining the periodontal regenerative capacity.

**Figure 1:**
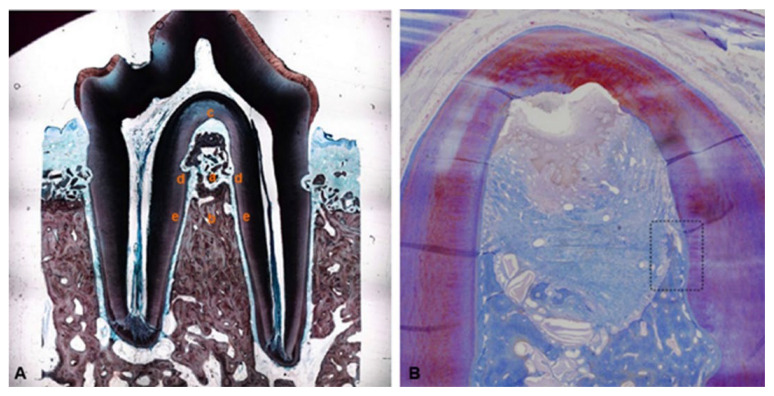
Cellular therapy in intrabony periodontal defects. (**A**) Dog cells derived from cementum: new alveolar bone formation (a), old alveolar bone (b), root notch (c), new cementum formation (d), and old cementum (e). (**B**) Dog mesenchymal cells derived from a periodontal ligament in which it is possible to observe: new alveolar bone formation, old alveolar bone, root notch, new cementum formation, and old cementum. Reproduced with permission from Nuñez et al. [64] under common creative license (CCC).

In cell therapy attempts, periodontal ligament stem cells (PDLSCs) have shown the potential to stimulate periodontal regeneration [59], unlike bone marrow stromal cells, which have a greater capacity for bone regeneration. PDLSCs, in addition to having similar characteristics to bone marrow stromal cells, including multipotency, self-renewal and immunomodulatory properties of the host response, also have a unique potential to regenerate the periodontal ligament [60]. They have also shown the ability to differentiate into cementoblasts, with increased alkaline phosphatase activity, increased matrix mineralization, and upregulated expression of mineralization-determining genes [61]. In addition, human PDLSCs, like bone marrow stem cells, have been shown to have a suppressive capacity for immune response and inflammatory reactions. PDLSCs are an excellent candidate for new allogeneic stem cell-based therapies [62]. This hypothesis was clarified in a study in a pig model in which transplanted allogeneic PDLSC did not show the presence of human leukocyte antigen (HLA)-II DR and similar cells. These results show that PDLSC possess minimal immunogenicity and increased immunosuppressive capacity through a T-cell-mediated pathway that induces the production of prostaglandin E2 [63]. In an experiment carried out on beagle dogs, the ability of cementum- and periodontal ligament-derived cells to promote regeneration in experimental models of chronic periodontal defects of critical intraosseous and supra-alveolar three-walled dimensions was demonstrated (Figure 1) [64].

Finally, in the results of these experimental studies on cell therapy, the key role of the use of an appropriate carrier, which ensures the viability and dynamism of the cells during the healing process, clearly emerges. Furthermore, for the type of regenerative technology used, selecting the type of vector and the appropriate soft tissue manipulation during regenerative surgery maintains the anatomy and structural characteristics of the tissue origin with anatomy that can promote tissue regeneration.

Initially, stem cells were implanted into the site to be regenerated using a periodontal regeneration scaffold, such as collagen, fibrin, hydrogel, and gelatin [65]. However, these delivery techniques were complex both during implantation at the site and in post-implantation management, as they were very prone to potential rejection by the host. To overcome these obstacles, research has gone into evaluating scaffold-free tissue delivery techniques [66].

In this regard, there are two scaffold-free strategies: cell injection and cell sheets [67]. The injection of cells is a common treatment in stem cell-based tissue engineering, which has also found remarkable efficacy in the treatment of periodontitis (local injection of dental pulp stem cells (DPSC) or PDLSC as well). In fact, in one study, periodontal regeneration was observed in a rat model of periodontitis after local injection of a bone marrow mesenchymal stem cell (BMMSC) suspension [68]. Cell sheet engineering is a unique and scaffold-free method of cell processing, exploiting ascorbic acid culture or using temperature-sensitive cell culture vessels. Compared to the previous technique described, cell sheets allow the preservation of the extracellular matrix (ECM) and cell–cell junctions, which would be degraded by proteolytic enzymes such as trypsin and/or dyspase [67].

PDLSCs possess low immunogenicity and marked immunosuppression via PGE2-induced T-lymphocyte anergy. They can induce macrophage polarization with respect to the M2 phenotype, which contributes to enhanced periodontal regeneration after stem cell transplantation [63].

Periodontal regeneration has been achieved in human studies using PDLSCs, BMMSCs, and cells derived from the gingiva or periosteum. Two studies were conducted for periodontal regeneration using PDLSCs. The first study evaluated the efficacy and safety of autologous PDLSCs for the regeneration of deep periodontal infrabony defects in 35 patients (NIH clinical trial registration number: NCT01357785). Autologous sheets of PDLSCs were transplanted into intrabony defects in association with freeze-dried bone and the sites were monitored for 12 months. No clinical safety issues were observed during these clinical studies. However, no statistically significant differences were observed between the group treated with PDLSC sheets and the control group. In the second study, the efficacy of periodontal regeneration by an allogeneic PDLSC cell sheet was evaluated in 80 patients (NIH clinical trial registration number: NCT01082822), but his result remains unknown. [67].

Genomic studies on periodontal ligament cells obtained from cultured cells have shown the great ability of periodontal ligament cells to obtain periodontal regeneration [55,69]. Cell lines from the periodontal ligament are described as a large number of cells associated with an abundant ECM, which contains collagen type I, fibronectin, and integrin-1 beta [55]. The results of in vitro experiments suggest that the periodontal ligament cells have a differentiation potential capable of regenerating periodontal tissues and alveolar bone. Recent studies have demonstrated that periodontal ligament cells co-cultured with IL-11 increase alkaline phosphatase activity in cells cultivated with dexamethasone [55,69]. This could represent a potential new method of periodontal regeneration to create new regenerative therapies for the periodontal ligaments that would allow transplanting periodontal ligaments with their preserved structures to be transplanted directly in vivo into tissues instead of being damaged by periodontal disease. These innovative therapies have been extended in their clinical use, and therefore cellular engineering research offers a substantial potential application in the future in the field of regenerative medicine [70].

## 5. Cell Signaling Strategies

Many authors have reported how strategies that aim to prevent cellular activation act to inhibit intracellular signals transduced when ligands bind their respective receptors on cell membranes. In addition to the action of cytokines, signal transduction pathways are also activated by environmental stress or some bacterial mediators, such as proteins, bacterial products, or lipopolysaccharide [19,71,72,73]. The activation of the signal transduction pathways brings an uptake of different transcription molecules and similar mediators that modulate the release of proteases, cytokines, proteases, and various other complexes that are activated during an inflammatory process [74]. Specifically, bacterial release products and cytokines upregulate several signal transduction pathways directly associated with the inflammatory phase, including the mitogen-activated protein kinase (MAPK), the phosphatidylinositol-3 protein kinase (PI3) pathway, and the Janus kinase signal pathway. MAPKs are protein kinases involved in the regulation of multiple cellular responses (proliferation, gene expression, differentiation, and apoptosis) to a wide range of stimuli, including mitogens, osmotic stress, terminus shock, and pro-inflammatory cytokines. We have provided more detail in the text on the function of MAPKs and their role in the inflammatory process triggered in periodontal disease. MAPKs are intimately involved in periodontitis, as stimulation by the PLS of *A. actinomycetecomitans* induces overregulation of this pathway, which contributes to the maintenance of the inflammatory environment and increased periodontal resorption through activation of the transcription of various inflammatory mediators [75]. Other inflammatory signal transduction methods concern the selectin and integrin immunoreceptors, G protein-coupled receptors (chemokines), and steroid hormone-derived receptors. So the modern therapeutic approaches adopted are aimed at these signaling pathways, especially MAPK and the nuclear factor kappa-light-chain-enhancer of activated B cells (NF-κB) [76].

The binding of the ligand to its membrane receptor, similar to the binding process of the lipopolysaccharide, causes a change in its conformation due to the phosphorylation of the receptor itself or of an associated enzyme [77]. Various phosphorylation processes, mediated by some protein kinases, lead to the cascade activation of different transcription factors or other proteins useful for post-transcriptional gene regulation thanks to different mRNAs that finally induce protein synthesis. Transcription factors are usually activated by phosphorylation obtained through specific protein kinases through a process of greater affinity to the transcription factor/DNA [78]. However, in order to better balance these phosphorylative phenomena, phosphatase kinase proteins (MKP) can use dephosphorylation mechanisms, thus counteracting their effects [79]. This process takes place in the post-transcriptional phase thanks to the presence of RNA-binding proteins (RNABPs, including TIA-1 and TIAR) that are able to bind to highly specific sequences, rich in adenosine-uridine (AURE), of mRNA. AURE elements are found in the 3TR (3TR) region of various pro-inflammatory cytokines (including TNF-α, IL-6, and COX-2), and reduce their protein synthesis, giving the molecules a specific instability of mRNA or by gene silencing translational. Indeed, some studies have shown that the phosphorylation of TIAR and TIA-1 has resulted in the inhibition of the degradation of mRNA and leading to higher protein production [80].

Furthermore, there are other mechanisms that regulate inflammatory signal transduction during different inflammatory pathways and on which one can act by exploiting the action of specific inhibitory proteins that inhibit ligand-receptor binding and negatively regulate inflammatory signal transduction [81].

The NF-kB pathway is important in pro-inflammatory signaling, inducing the release of cytokines, chemokines, and adhesion molecules. Increased NF-κB activation in tissues at the site of injury from patients with periodontitis indicates an important pathogenic role of NF-κB in periodontitis. *P. gingivalis* virulence factors induce activation of the NF-kB pathway and induce inflammation through toll-like receptors [82]. In this regard, modern therapeutic targets that provide for the transduction pathway of the NF-κB signal have aroused interest. The transcription factors NF-κB and the protein-1 activator regulate, at the periodontal level, the expression of various inflammatory mediator tracks such as collagenases, TNF-α, IL-1, IL-6, chemokines, and the different molecules of membership intercellular cell adhesion molecule (ICAM) and vascular cell adhesion molecule (VCAM) [83,84,85].

The transcription factors NF-κB consist of homo- or heterodimers normally present in the cytoplasm of most cells in humans. The NF-κB family includes NF-κB1 (p50), -κB2 (p52), p65 (RelA), RelB, and c-Rel. Various in vitro models have shown that different periodontal pathogenic bacteria, including *P. gingivalis*, are able to periodically activate the NF-κB pathway, especially during the active inflammatory phases [3,86]. The pro-inflammatory cytokines IL-1 and TNF and the various bacterial lipopolysaccharides are present in high quantities during the active periodontal period and are able to activate the NF-κB pathway. Immunohistochemistry studies have shown greater expression in sites with periodontitis compared to healthy sites for NF-κB production (p50/p65) [87]. Furthermore, it has been shown that, in the absence of inflammation, the nuclear translocation of NF-κB is prevented by the κB inhibitor protein with which NF-κB is coupled. So when there is an inflammatory stimulus induced by a cytokine, the cascade recruitment of other pro-inflammatory cytokines, including the TRAF protein, determines the activation of the enzyme NF-κB-inducing kinase (NIK), which is therefore essential to inhibit the mediated NF-κB inflammatory pathway [88].

Similarly to NF-κB, MAPKs are signal transduction pathways comprising p38, NK, and signal-regulated extracellular kinases (ERK 1/2) [89]. Mitogenic agents and various growth factors primarily activate the ERK1/2 forms; otherwise, p38 and JNK are usually activated by IL-1 and TNF-α and by factors that determine cellular stress, including thermal shock proteins and free radicals derived from oxygen [90]. It is believed that the various MAPK families are present with gingival tissues during periodontitis, even if their level of expression may change based on the severity of the inflammation and on the type of tissue involved [91]. In periodontal tissue, MAPKs are essentially IL-1, TNF-α, and lipopolysaccharide [92]. The MAPK p38a isoform is among the main ones to be activated intracellularly during inflammatory stages. Through the stabilization of mRNA and direct activation of gene transcription, the p38a isoform is able to stimulate the production of IL-1, IL -6, IL-8, and TNF-α [92]. In addition, it has been shown that p38 controls the production of other mediators, including prostaglandins, metalloproteases, and chemokines [93,94].

Anti-cytokine biological therapy’s effectiveness mainly demonstrates the biological theory that a reduction in the release of a cytokine can slow down an inflammatory process in patients with inflammatory bone diseases. Recently, the identification of new pro-inflammatory signal transduction pathways has allowed the development of a new and very interesting therapeutic target [95,96]. Currently, in accordance with all the new knowledge acquired in the field of the pathogenesis of the periodontal disease, several biological drugs used for the treatment of other chronic inflammatory and autoimmune diseases, such as rheumatoid arthritis and Crohn’s disease, are being evaluated. Although these drugs have some benefits, they are poorly targeted for periodontal disease, are expensive and may induce numerous side effects that far outweigh the benefits induced [97]. Currently, studies on human samples evaluating the activity of anti-cytokine and immune-modulating molecules are scarce, as they are very invasive drugs. These molecules are currently widely used in the treatment of rheumatoid arthritis and other autoimmune diseases [30]. Rheumatoid arthritis has several aspects in common with periodontal disease, which supports the idea that these drugs could be used in the treatment of periodontitis [98]. Holding back their use are the countless undesirable effects. In one study, it was identified that patients with rheumatoid arthritis receiving intravenous infliximab had lower bleeding on probing scores, lower concentrations of tumor necrosis factor-alpha in gingival crevicular fluid, and lower mean probing depth measurements than patients with rheumatoid arthritis who were not receiving antitumor necrosis factor alpha therapy and with non-rheumatoid arthritis controls [99]. A subsequent study of three groups of patients (those with autoimmune diseases, including rheumatoid arthritis who were not receiving antitumor necrosis factor-alpha therapy; those with rheumatoid arthritis who were receiving antitumor necrosis factor-alpha therapy; and healthy controls without autoimmune diseases and no anti-TNF-α therapy) identified that patients with autoimmune diseases who were not receiving antitumor necrosis factor alpha therapy had more gingival inflammation, more bleeding on probing, higher mean probing depth and higher gingival crevicular fluid concentrations of antitumor necrosis factor-alpha than patients in the other two groups [100]. Both studies had a major limitation: at baseline, patients with autoimmune diseases (including rheumatoid arthritis) have more periodontal inflammation than patients without autoimmune diseases and that administering anti- TNF-α therapy as a treatment for rheumatoid arthritis reduces inflammation in periodontal tissues [99,100]. Therefore, these preliminary results will have to be translated into future scientific efforts, which will have to better analyze the inflammatory signal transduction pathways for early treatment of periodontitis from its initial stages.

## 6. Mediators against Bone Resorption

**Figure 2:**
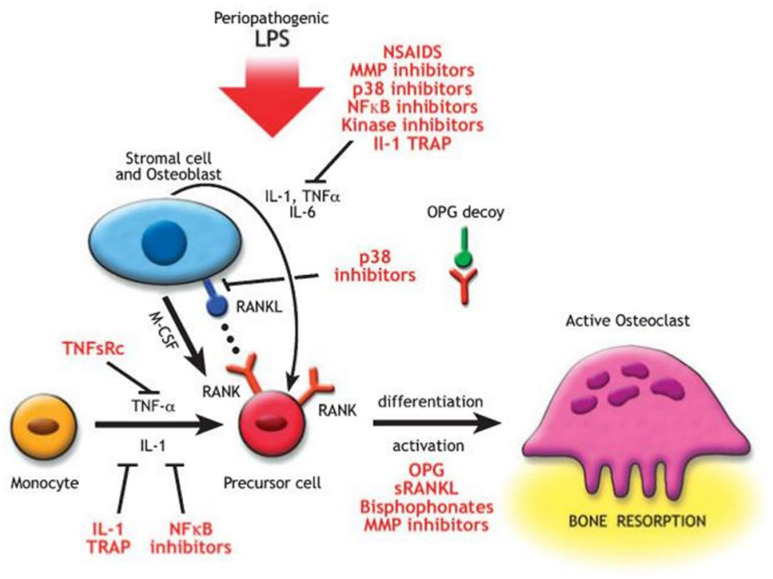
Agents that block the differentiation or activity of osteoclasts are potential therapeutic strategies to treat bone resorption. OPG inhibits the differentiation of osteoclasts through its action as a decoy receptor that blocks the receptor activator of nuclear factor-kappa B (NF-κB) ligand (RANKL) and RANK juxtacrine interaction. Non-steroidal anti-inflammatory drugs (NSAIDs) and other anti-inflammatory molecules can inhibit the formation of hematoprogenitor cells to pre-osteoclasts. Matrix metalloproteinase (MMP) inhibitors reduce the protease degradation of the organic matrix, and anti-integrins block the initial osteoclast adhesion to the matrix. IL, interleukin; LPS, lipopolysaccharide; M-CSF, macrophage colony-stimulating factor; sRANKL, soluble RANKL; TNF-α, tumor necrosis factor-a; TNFsRC, TNF soluble receptor. Reproduced with permission from Kirkwood et al. [85], under common creative license (CCC).

Among the main mediators against periodontal disease, various factors can be counted, e.g., hormones (parathyroid hormone, vitamin D3, and proteins related to parathyroid hormones), TNF-α, IL-1, -6, -11, and -17, growth factors (bone-2 morphogenetic protein, BMP) and other mediators such as prostaglandin E2, CD40 ligand activating T (CD40L), and glucocorticoid cells [101]. These mediators have been demonstrated to stimulate the production and release of the receptor activator of the nuclear factor kappa-Β ligand (RANKL) gene in stromal cells and osteoblasts [102]. RANKL also regulates the stimulation of osteoclastogenesis across the interaction with the receptor activator of nuclear factor kappa-Β (RANK) expressed on pre-osteoclastic lines. The interaction between RANKL and RANK promotes these cells’ maturation, allowing osteoclasts to be activated. Instead, osteoprotegerin, expressed by osteoblastic cells, acts as a trick-receptor, since binding to RANKL inhibits the development of osteoclasts [85]. Furthermore, it has been shown that osteoprotegerin, when expressed by osteoblasts, acts as if it were a trick receptor that binds to RANKL and thus inhibits the development of osteoclasts [22,85] (Figure 2).

Previous studies have shown that T lymphocytes may be able to regulate mechanisms of inflammation and bone resorption in alveolar bone [103]. More specifically, in a study by Teng et al. [26], conducted on mice deprived of endogenous B and T lymphocytes and inoculated with strains of *A. actinomycetemcomitans*, it was observed that the bacteria that activated the CD4+ T-cell reaction, in turn, led to an upregulation of RANKL and subsequent destruction and resorption of alveolar bone [104]. The results of these studies indicate that RANKL, activated by CD4+ T cells following periodontal bacterial infection, played an important role in alveolar bone destruction [10]. It has been observed that the RANKL/osteoprotegerin ratio increases in patients with periodontitis compared to healthy subjects, indicating that the RANKL/osteoprotegerin interaction may be critical in modulating alveolar bone loss during periodontitis [24,105]. For these reasons, the use of osteoprotegerin was evaluated as a modulator of alveolar bone loss during periodontitis [106]. In accordance with this theory, an animal model study demonstrated that the inhibition of RANKL/osteoprotegerin, in mice orally infected with *A. actinomycetemcomitans*, resulted in a significant reduction in the osteoclasts’ activity and alveolar bone loss [107]. The mRNA expression of RANKL and OPG was also studied in the gingival tissues of patients with gingivitis, chronic periodontitis, and generalized aggressive periodontitis before non-surgical periodontal treatment [25,108,109].

It was shown that RANKL was expressed in all three forms of periodontitis but not in gingivitis, and the most obvious increase was observed in generalized aggressive periodontitis [110].

The interactions between *P. gingivalis*, RANKL, and OPG are important in the pathogenesis of periodontitis. Bostanci et al. [111] showed that the upregulation of RANKL mRNA and increased RANKL/OPG mRNA ratio was associated in clinically human gingival tissues of patients with periodontitis. In this regard, it has been shown *P. gingivalis* may modulate the RANKL/OPG expression in periodontal tissues by direct or indirect mechanisms and stimulate osteoclastogenesis, leading to alveolar bone resorption. All tissues expressed OPG, and its levels were decreased in chronic periodontitis compared to in gingivitis and healthy samples [111].

RANKL expression was significantly correlated with increased numbers of *P. gingivalis* [112]. *P. gingivalis*, and most likely its lipopolysaccharide, regulates the RANKL-OPG system pathway prostaglandin E2, promoting osteoclastogenesis and contributing to the bone loss observed in periodontitis [113]. However, local OPG gene transfer has been shown to inhibit LPS-induced alveolar bone resorption [16].

Gao et al. conducted a study in which they administered vitamin D supplementation to 360 patients with moderate to severe periodontitis after non-surgical periodontal therapy (NSPT). Patients were randomly assigned to 2000 international units (IU)/day of vitamin D3, 1000 IU/day of vitamin D3, or placebo. The effect of vitamin D supplementation tended to be modest, with limited periodontal clinical relevance and long-term efficacy against PPD and CAL [114].

In addition, Perayil et al. evaluated, in 82 patients with moderate chronic periodontitis, the effects of vitamin D plus calcium supplementation (Shelcal-D 500 mg calcium + 250 IU vitamin D once daily) compared to placebo after non-surgical periodontal therapy. Although PPD and CAL did not differ between the groups, the authors reported significantly better results for the vitamin D group in relation to gingival inflammation and bone density (measured by panoramic radiography) [115].

In conclusion, based on this preclinical evidence, the RANK-RANKL-osteoprotegerin interactions and modulations may constitute an emerging therapeutic mediator for the treatment of periodontitis and alveolar bone loss, especially during the active phases of the disease.

## 7. Enamel Matrix Derivatives

Enamel matrix derivatives (EMDs) are now an extremely well-known and quite commonly used product in the tissue regeneration of periodontal bone defects. A number of studies have evaluated the effects of EMDs on human periodontal regeneration. EMDs consist of a mixture of candidate proteins, which have been shown to stimulate the differentiation of mesenchymal cells into the periodontal cell complex [116,117]. The regenerative action of EMDs can be attributed to the interference of osteoclastic activation. In fact, an increase in the production of osteoprotegerin (OPG), which interferes with the formation of the RANK-RANKL complex, and a reduced synthesis of RANKL at the osteoblastic level have been associated with the action of EMDs [118]. In addition, EMDs are said to stimulate proliferation and, at the same time, inhibit apoptosis of fibroblasts, which are responsible for the release of the ECM. The vascular endothelial growth factor (VEGF) increase would facilitate tissue oxygenation, inducing angiogenesis. Lastly, TGF-β, a fundamental molecule in the organization of tissue repair, appears to be overexpressed in gingival fibroblasts and periodontal ligament cells through the action of the EMD [119]. Two systematic reviews that investigated enamel matrix derivatives in periodontal therapy concluded that enamel matrix could both significantly improve clinical attachment levels and result in reduced pocket probing depth compared to open flap debridement alone [120,121]. The EMD appears to positively influence PDL cells, cementoblasts, and osteoblasts while inhibiting epithelial cells—a characteristic favorable for the re-establishment of the periodontal tissues [122]. Another review on this topic, which only analyzed studies conducted on human samples, suggests that, although several studies have been conducted to evaluate the efficacy of the EMD in terms of periodontal wound healing and complications, further investigation is needed [123]. Current results from human studies have indicated that the EMD may play an important role in periodontal wound healing in terms of fewer postsurgical complications than guided tissue regeneration (GTR) and improved incision healing by promoting the formation of blood vessels and collagen fibers in the connective tissue [124]. In addition, clinical studies have indicated that EMD treatment positively influences periodontal wound healing after surgical treatment [125].

## 8. Platelet-Rich Plasma

In addition to matrix derivatives, autologous platelet-rich plasma is another useful material for promoting periodontal regeneration, obtained by separating and concentrating platelets from the blood [126,127]. The application of platelet concentrates was initially limited to treating and preventing hemorrhage due to severe thrombopenia. However, with medical advances in recent decades, platelet concentrates have been proposed as usable compounds. As the scope in medical application has expanded, it has been attempted to combine the sealing properties of fibrin with growth factors in platelets for wound healing and tissue regeneration, thanks to the sealing properties and growth factors contained in platelets. In detail, platelets contain biologically active proteins that bind to a developing fibrin network or ECM. The proteins thus create a chemotactic gradient for the recruitment of stem cells, which differentiate and promote healing through regeneration [128]. Several clinical studies have been carried out in vitro and on animal and human heads. Protein-rich plasma is obtained from centrifuged blood without any added anticoagulant or bovine thrombin. The resulting product consists of the following three layers: top layer consisting of an acellular plasma, platelet-rich fibrin (PRF) clot in the middle (which is the part to be used in the treatment of periodontal defects), and a red corpuscular base at the bottom [128]. Several studies have indicated that the use of platelet-rich plasma, used alone or with a combined guided tissue regeneration technique, led to improvements, although not statistically significant, in clinical attachment loss (CAL) and probing depth (PD) in patients with intrabony defects [129,130]. A recent study evaluated and compared the efficacy of PRF with enamel matrix derivative (EMD; Emdogain) in the treatment of periodontal intrabony defects in patients with chronic periodontitis six months after surgery. In the study, 44 periodontal patients with intrabony defects were enrolled and divided into two groups of 22. Each group was randomly assigned a treatment: EMD (n = 22) and PRF (n = 22). Postsurgical measurements showed an equal reduction in probing depth and a greater but statistically insignificant gain of attachment for the Emdogain group compared to the platelet-rich group with fibrin. The Emdogain group had a significantly greater percentage of defect resolution (43.07% ± 12.21) than the platelet-rich fibrin group (32.41% ± 14.61). As the study shows, both Emdogain and platelet-rich fibrin were effective in regenerating intrabony defects. Emdogain was significantly superior in terms of the percentage resolution of defects [131]. Similar results were also reported in a recent meta-analysis comparing the results of treating periodontal intrabony defects in several RCT studies using platelet-rich fibrin (PRF) with other commonly used modalities. The results showed that the use of OFD/PRF statistically significantly reduced PD and improved CAL and RBF compared to OFD. No clinically significant differences were reported when OFD/BG was compared to OFD/PRF. The addition of PRF to OFD/BG led to significant improvements in CAL and RBF. No differences were reported between any of the following groups (OFD/BM, OFD/PRP and OFD/EMD) compared to OFD/PRF. Additionally, no improvements were reported when PRF was added to OFD/EMD. The addition of all three of the following biomolecules (metformin, bisphosphonates, and statins) to OFD/PRF resulted in statistically significant improvements in PD, CAL, and RBF. Therefore, the use of PRF significantly improved clinical outcomes in intrabony defects compared to OFD alone with similar levels observed between OFD/BG and OFD/PRF [132]. Therefore, these results suggest that the regenerative effects of platelet-rich plasma are not yet well understood and require further clinical studies to validate their clinical efficacy in association with future research investigating PRF at the histological level.

## 9. Growth Factors

Of the growth factors, platelet-derived growth factor (PDGF), a dimer composed of peptide chains A and B, was of considerable interest. Of these, the homodimer BB isoform has been shown to be a potent mediator of periodontal regeneration by strongly accelerating wound healing and stimulating the proliferation of periodontal ligament cells [133]. Lynch et al. demonstrated that PDGF, in a study of beagle dogs, was useful in achieving periodontal regeneration when combined with insulin growth factor (IGF) [134]. Accordingly, Rutherford et al. showed favorable results of PDGF in its ability to stimulate periodontal tissue regeneration in monkeys. However, the use of PDGF has not yet been licensed in several countries, and further longitudinal clinical data are needed to confirm the potential regenerative efficacy of this growth factor [135].

Bone morphogenetic protein-2 (BMP-2) is a homodimer characterized by a disulfide bridge that has been shown to play an important role in osteoblast differentiation [136]. The production of BMP-2 can be achieved through specific recombinant DNA technology. Some studies [137,138,139] have observed the possibility of achieving periodontal regeneration through the use of recombinant human BMP-2 with different types of carriers. On the other hand, adverse effects such as root resorption have been reported, and therefore BMP-2 has not yet been considered fully valid for periodontal regeneration [140,141].

Cement-derived growth factor has been reported to have the ability to stimulate periodontal regeneration, and its effect is shown through the modulation of genetic pathways to achieve tissue regeneration through the induction of alkaline phosphatase (ALP) activity [142]. Human cementum protein 1 (CEMP1), known to induce the differentiation of cementoblasts and osteoblasts, also greatly influences ALP activity in human periodontal ligament-derived cells in vitro and promotes bone regeneration in vivo. Indeed, it has been observed that the expression of CEMP1 can stimulate differentiation toward a “mineralizing” cell phenotype, and its increase is also associated with continued cementum growth. In detail, a recent study indicated that CEMP1 may have a direct role in promoting the proliferation and differentiation of human oral mucosal stem cells toward a “mineralizing” phenotype by activating the β-catenin signaling cascade [143]. Proteins such as OCN, BSP, and OPN have affinity for the mineral phase that controls nucleation; crystal growth and mineral maturation of the apatite crystals are also expressed [144]. Maximum levels of BSP expression occur during the initial stages of mineralization and diminish at the onset of mineralization [145]. Studies have also shown that inflammatory mediators influence the regeneration of periodontal tissues containing cementoblasts [146]. Similarly, Pham et al. demonstrated that prostanoids induce the release of gene-1 (egr-1), which can regulate anabolic responses in a mouse model [147]. Kajiya et al. [148] demonstrated that brain-derived neurotrophic factor (BDNF) could determine, in human cement cells, the expression of genes that stimulate bone and root cement production through the production of certain mitogen-active protein kinases [149]. BDNF activates a TrkB-c-Raf-ERK1/2-Elk-1 signaling pathway in human cementoblast-like cell lines (HCEM) cells. The culmination of these signaling events is the upregulation of the mRNA expression of ALP, OPN, and BMP-2. These observations provide critical new insights into the mechanisms whereby BDNF promotes the regeneration of periodontal tissue and suggest that ERK1/2 signaling contributes to the enhancement in the formation of mineralized tissues, such as cementum and bone [150]. All these studies suggest that the use of growth factors can lead to good long-term results for periodontal regeneration.

## 10. Translating Epigenetics during Periodontitis

Genome-wide association studies have made it possible to identify genetic alternatives for the treatment of diseases in humans. During periodontitis, epigenetic changes induced by periodontopathogens and their release mediators, such as lipopolysaccharides, can be found.

Several in vitro studies have shown important changes in CpG island of DNA methylation [151]. Both hyper- and hypomethylation have been found in various cellular constituents following infection with periodontal pathogens, and subsequent cascading effects on gene regulation depend on the cellular interaction of the host with specific pathogenic bacteria [151,152].

An association between DNA methylation and impaired immune system function is now well established, as DNA methylation plays an essential role in the regulation of inflammatory genes. It has been shown that the epigenome itself is influenced by inflammation. Therefore, epigenetics has become essential in producing and releasing several pro-inflammatory mediators, including IL-1, IL-6, TNF-α and interferon-gamma, as well as in the induction of NF-κB and cyclooxygenase 2 [152].

In this regard, periodontitis has been correlated with altered methylation levels of genes related to the associated inflammatory response [153] in subjects with both periodontitis and gingivitis (Table 1).

Furthermore, it has been proposed that a discrepancy in the ratio of antioxidants to reactive oxygen species (ROS) is involved in the etiology of periodontal disease and its treatment [154]. One mechanism by which ROS are involved in periodontal pathogenesis is through the activation of inflammasomes. ROS induce activation of the pyrin domain of the NOD-like receptor (NLR) type 3 family, which can result in characteristic tissue damage during periodontitis [155]. The pyrin domain of the NLR, comprising three inflammasome loops, allows the interaction of the protein with oxidative stress and the release of IL-1β, IL-17, and IL 23 in response to metabolic stress [32,156].

Of recent importance is the finding that certain histone deacetylase inhibitors appear to be able to regulate the effects of RANKL on osteoclasts and inflammatory cells, resulting in the inhibition of RANKL-induced osteoclastic activity in mouse macrophages and murine cell lines [157,158]. In human studies, histone deacetylase inhibitors inhibited osteoclastic bone resorption in vitro, probably due to reduced expression of osteoclast transcription factors such as tumor necrosis factor receptor-associated factor 6, activated T lymphocyte nuclear factor, and osteoclast-associated receptor during late osteoclast differentiation. These results show that histone deacetylase inhibitors may be promising as new therapies in the treatment of diseases characterized by bone resorption, such as periodontitis [159]. However, undesirable effects are certainly possible due to the wide-ranging effects of these molecules in various tissues and cells, so topical application could be useful to reduce systemic exposure, and periodontitis could be a useful model to further investigate this premise.

In addition, a study in diabetic patients with periodontitis showed that DNA methylation was one of the regulators of proteins interacting with thioredoxin, highlighting possible new target therapies for type 2 diabetes and a reflected impact in the control of diabetes-associated periodontitis [160].

Although there are real limitations of current therapies to date, epigenetic therapies represent a real, achievable goal for the preventive treatment of complex diseases that would also lead to a reduction in associated economic costs. However, a better understanding of the complexity of the epigenome, its methylations, and its role in gene therapy on periodontitis-related environmental factors are needed to design target therapies [161]. In fact, the discovery of the key role of epigenetic modifications associated with complex diseases and in cancer therapy has aroused great interest from the scientific community in the development of new drugs aimed at interfering with epigenetics in order to restore a physiological and epigenetic horizon to characterize disease prevention through epigenetic target therapies aimed at each patient.

## 11. Conclusions

In light of the scientific evidence reviewed in this review, it can be emphasized that the response to therapy of periodontal patients is not always predictable, as it is influenced by both the composition of the periodontal biofilm and the interindividual host immune response. This awareness has prompted the search for treatments that can be targeted to the characteristics of the microbiota profile and patient-specific response, leveraging the new immune-modulatory and regenerative therapies that have emerged in the treatment of periodontal disease during recent years. Although numerous animal and in vitro studies, as well as patient studies, have been conducted, to date most of the research on the use of these new therapeutic strategies has been limited to small clinical trials, the results of which are not yet fully consistent. This can be attributed to small sample sizes and the lack of standardized protocols, especially in humans. The considerable interest in anticytokinetic drugs and other categories of drugs that act by modulating inflammation in the treatment of periodontitis has been somewhat dampened by the realization that molecules currently on the market can induce numerous side effects. In the coming years, therefore, research will focus on studying new molecules or revising current ones to reduce unwanted effects and favor those with a useful impact on periodontal disease.

## Figures and Tables

**Table 1:** Target genes of DNA methylation during periodontitis.

Target Genes	Tissues	Epigenetic Dynamics	Mechanisms
Interleukin-8	Epithelial oral cells	The frequency of methylation of the interleukin-8 gene promoter was found to be higher in controls than periodontitis	Increased levels of interleukin-8
Tumor necrosis factor	Gingival biopsies	Two CpG sites within the tumor necrosis factor promoter displayed increased methylation in chronic periodontitis compared with gingival health	Decreased levels of tumor necrosis factor
Interleukin-17c	Gingival biopsies	CpG methylation of interleukin-17C reduced in patients with aggressive periodontitis compared with healthy individuals	Increased levels of interleukin-17C
Interleukin-6	Serum and mononuclear cells	The -74 bp CpG site within the interleukin-6 promoter displayed hypomethylation in samples from periodontitis patients than in controls	Increased levels of interleukin-6
Interferon-gamma	Gingival biopsies	The methylation levels in samples collected from periodontitis subjects were found to be lower than in healthy tissues	Increased levels of interferon-gamma
Cyclooxygenase-2	Blood samples, oral epithelial cells and gingival biopsies	Higher methylation levels of cyclooxygenase 2 were found in samples from patients with periodontitis in comparison to controls	Decreased expression of the cyclooxygenase-2 gene and the levels of cyclooxygenase 2 protein
Toll-like receptor 2	Gingival biopsies	Higher methylation levels of toll-like receptor 2 were found in samples from patients with periodontitis than in controls	Decreased levels of toll-like receptor 2

## Data Availability

Data are available from the corresponding author upon reasonable request.

## References

[1] Tonetti M.S., Greenwell H., Kornman K.S. (2018). Staging and grading of periodontitis: Framework and proposal of a new classification and case definition. J. Periodontol..

[2] Pan W., Wang Q., Chen Q. (2019). The cytokine network involved in the host immune response to periodontitis. Int. J. Oral Sci..

[3] Sugita N., Kimura A., Matsuki Y., Yamamoto T., Yoshie H., Hara K. (1998). Activation of transcription factors and IL-8 expression in neutrophils stimulated with lipopolysaccharide from Porphyromonas gingivalis. Inflammation.

[4] Olsen I., Chen T., Tribble G.D. (2018). Genetic exchange and reassignment in Porphyromonas gingivalis. J. Oral Microbiol..

[5] Olsen I., Singhrao S.K. (2020). Is there a link between genetic defects in the complement cascade and Porphyromonas gingivalis in Alzheimer’s disease?. J. Oral Microbiol..

[6] Olsen I., Singhrao S.K. (2018). Importance of heterogeneity in Porhyromonas gingivalis lipopolysaccharide lipid A in tissue specific inflammatory signalling. J. Oral Microbiol..

[7] Olsen I., Yilmaz Ö. (2019). Possible role of Porphyromonas gingivalis in orodigestive cancers. J. Oral Microbiol..

[8] Ebbers M., Lübcke P.M., Volzke J., Kriebel K., Hieke C., Engelmann R., Lang H., Kreikemeyer B., Müller-Hilke B. (2018). Interplay between *P. gingivalis*, *F. nucleatum* and *A. actinomycetemcomitans* in murine alveolar bone loss, arthritis onset and progression. Sci. Rep..

[9] Graves D.T., Jiang Y., Valente A.J. (1999). The expression of monocyte chemoattractant protein-1 and other chemokines by osteoblasts. Front. Biosci..

[10] Yan K., Lin Q., Tang K., Liu S., Du Y., Yu X., Li S. (2020). Substance P participates in periodontitis by upregulating HIF-1α and RANKL/OPG ratio. BMC Oral Health.

[11] Ye D., Gajendra S., Lawyer G., Jadeja N., Pishey D., Pathagunti S., Lyons J., Veazie P., Watson G., McIntosh S. (2020). Inflammatory biomarkers and growth factors in saliva and gingival crevicular fluid of e-cigarette users, cigarette smokers, and dual smokers: A pilot study. J. Periodontol..

[12] Graves D., Oskoui M., Voleinikova S., Naguib G., Cai S., Desta T., Kakouras A., Jiang Y. (2001). Tumor necrosis factor modulates fibroblast apoptosis, PMN recruitment, and osteoclast formation in response to P. gingivalis infection. J. Dent. Res..

[13] Uchida M., Shima M., Shimoaka T., Fujieda A., Obara K., Suzuki H., Nagai Y., Ikeda T., Yamato H., Kawaguchi H. (2000). Regulation of matrix metalloproteinases (MMPs) and tissue inhibitors of metalloproteinases (TIMPs) by bone resorptive factors in osteoblastic cells. J. Cell. Physiol..

[14] Olsen I. (2018). Relationship between serine dipeptide lipids of commensal Bacteroidetes and atherosclerosis. J. Oral Microbiol..

[15] Olsen I. (2018). Organization of supragingival plaque at the micron scale. J. Oral Microbiol..

[16] Olsen I., Singhrao S.K., Potempa J. (2018). Citrullination as a plausible link to periodontitis, rheumatoid arthritis, atherosclerosis and Alzheimer’s disease. J. Oral Microbiol..

[17] Olsen I., Yamazaki K. (2019). Can oral bacteria affect the microbiome of the gut?. J. Oral Microbiol..

[18] Santonocito S., Palazzo G., Indelicato F., Chaurasia A., Isola G. (2022). Effects induced by periodontal disease on overall quality of life and self-esteem. Mediterr. J. Clin. Psychol..

[19] Rogler G., Biedermann L., Scharl M. (2017). Anti-cytokine strategies beyond anti-tumour necrosis factor-α therapy: Pathophysiology and clinical implications. Dig. Dis..

[20] Monasterio G., Budini V., Fernández B., Castillo F., Rojas C., Alvarez C., Cafferata E.A., Vicencio E., Cortés B.I., Cortez C. (2019). IL-22-expressing CD4(+) AhR(+) T lymphocytes are associated with RANKL-mediated alveolar bone resorption during experimental periodontitis. J. Periodontal. Res..

[21] Aubin J.E., Bonnelye E. (2000). Osteoprotegerin and its ligand: A new paradigm for regulation of osteoclastogenesis and bone resorption. Osteoporos. Int..

[22] Hofbauer L.C., Heufelder A.E. (2000). The role of receptor activator of nuclear factor-κB ligand and osteoprotegerin in the pathogenesis and treatment of metabolic bone diseases. J. Clin. Endocrinol. Metab..

[23] Kantarci A., Van Dyke T.E. (2005). Lipoxin signaling in neutrophils and their role in periodontal disease. Prostaglandins Leukot. Essent. Fat. Acids.

[24] Matarese G., Currò M., Isola G., Caccamo D., Vecchio M., Giunta M.L., Ramaglia L., Cordasco G., Williams R.C., Ientile R. (2015). Transglutaminase 2 up-regulation is associated with RANKL/OPG pathway in cultured HPDL cells and THP-1-differentiated macrophages. Amino Acids.

[25] Liu D., Xu J., Figliomeni L., Huang L., Pavlos N., Rogers M., Tan A., Price P., Zheng M. (2003). Expression of RANKL and OPG mRNA in periodontal disease: Possible involvement in bone destruction. Int. J. Mol. Med..

[26] Teng Y.-T.A., Nguyen H., Gao X., Kong Y.-Y., Gorczynski R.M., Singh B., Ellen R.P., Penninger J.M. (2000). Functional human T-cell immunity and osteoprotegerin ligand control alveolar bone destruction in periodontal infection. J. Clin. Investig..

[27] Apatzidou D.A., Kinane D.F. (2010). Nonsurgical mechanical treatment strategies for periodontal disease. Dent. Clin..

[28] Ehmke B., Moter A., Beikler T., Milian E., Flemmig T.F. (2005). Adjunctive antimicrobial therapy of periodontitis: Long-term effects on disease progression and oral colonization. J. Periodontol..

[29] Jhinger N., Kapoor D., Jain R. (2015). Comparison of Periochip (chlorhexidine gluconate 2.5 mg) and Arestin (Minocycline hydrochloride 1 mg) in the management of chronic periodontitis. Indian J. Dent..

[30] Santonocito S., Indelicato F., Polizzi A., Palazzo G. (2021). Impact of periodontitis and orthodontic treatment on dental anxiety and self-esteem. Mediterr. J. Clin. Psychol..

[31] Yang B., Pang X., Li Z., Chen Z., Wang Y. (2021). Immunomodulation in the Treatment of Periodontitis: Progress and Perspectives. Front. Immunol..

[32] Zhou J., He Z., Yang Y., Deng Y., Tringe S.G., Alvarez-Cohen L. (2015). High-throughput metagenomic technologies for complex microbial community analysis: Open and closed formats. mBio.

[33] Roostalu J., Surrey T. (2017). Microtubule nucleation: Beyond the template. Nat. Rev. Mol. Cell Biol..

[34] Wrighton K.C., Thomas B.C., Sharon I., Miller C.S., Castelle C.J., VerBerkmoes N.C., Wilkins M.J., Hettich R.L., Lipton M.S., Williams K.H. (2012). Fermentation, hydrogen, and sulfur metabolism in multiple uncultivated bacterial phyla. Science.

[35] Reiman E.M., Langbaum J.B., Tariot P.N. (2010). Alzheimer’s prevention initiative: A proposal to evaluate presymptomatic treatments as quickly as possible. Biomark. Med..

[36] Nibali L., Henderson B., Tariq Sadiq S., Donos N. (2014). Genetic dysbiosis: The role of microbial insults in chronic inflammatory diseases. J. Oral Microbiol..

[37] Rose T.E., Zirkel P. (2007). Orton-Gillingham methodology for students with reading disabilities: 30 years of case law. J. Spec. Educ..

[38] Zarco M., Vess T., Ginsburg G. (2012). The oral microbiome in health and disease and the potential impact on personalized dental medicine. Oral Dis..

[39] Chen F.M., Shelton R.M., Jin Y., Chapple I.L. (2009). Localized delivery of growth factors for periodontal tissue regeneration: Role, strategies, and perspectives. Med. Res. Rev..

[40] Renvert S., Persson G.R. (2004). Supportive periodontal therapy. Periodontol 2000.

[41] Trombelli L. (2005). Which reconstructive procedures are effective for treating the periodontal intraosseous defect?. Periodontology 2000.

[42] Huang Q., Huang X., Gu L. (2021). Periodontal Bifunctional Biomaterials: Progress and Perspectives. Mater..

[43] Darby I.B., Morris K.H. (2013). A systematic review of the use of growth factors in human periodontal regeneration. J. Periodontol..

[44] Nuñez J., Vignoletti F., Caffesse R.G., Sanz M. (2019). Cellular therapy in periodontal regeneration. Periodontol 2000.

[45] Blufstein A., Behm C., Gahn J., Uitz O., Naumovska I., Moritz A., Rausch-Fan X., Andrukhov O. (2019). Synergistic effects triggered by simultaneous Toll-like receptor-2 and-3 activation in human periodontal ligament stem cells. J. Periodontol..

[46] Nakahara T. (2006). A review of new developments in tissue engineering therapy for periodontitis. Dent. Clin..

[47] Taba M., Jin Q., Sugai J., Giannobile W. (2005). Current concepts in periodontal bioengineering. Orthod. Craniofacial Res..

[48] Nakahara T., Nakamura T., Kobayashi E., Kuremoto K.-I., Matsuno T., Tabata Y., Eto K., Shimizu Y. (2004). In situ tissue engineering of periodontal tissues by seeding with periodontal ligament-derived cells. Tissue Eng..

[49] Xu J., Wang W., Kapila Y., Lotz J., Kapila S. (2009). Multiple differentiation capacity of STRO-1+/CD146+ PDL mesenchymal progenitor cells. Stem Cells Dev..

[50] Sonoyama W., Seo B.-M., Yamaza T., Shi S. (2007). Human Hertwig’s epithelial root sheath cells play crucial roles in cementum formation. J. Dent. Res..

[51] Liu Y., Zheng Y., Ding G., Fang D., Zhang C., Bartold P.M., Gronthos S., Shi S., Wang S. (2008). Periodontal ligament stem cell-mediated treatment for periodontitis in miniature swine. Stem Cells.

[52] Horiuchi K., Amizuka N., Takeshita S., Takamatsu H., Katsuura M., Ozawa H., Toyama Y., Bonewald L.F., Kudo A. (1999). Identification and characterization of a novel protein, periostin, with restricted expression to periosteum and periodontal ligament and increased expression by transforming growth factor β. J. Bone Miner. Res..

[53] Yamamiya K. (2008). Tissue-engineered human cultured periosteum sheets combined with platelet-rich plasma and porous hydroxyapatite graft in treating human periodontal infrabony osseous defects: A comparative controlled clinical study. J. Periodontol..

[54] Kawaguchi H., Hirachi A., Hasegawa N., Iwata T., Hamaguchi H., Shiba H., Takata T., Kato Y., Kurihara H. (2004). Enhancement of periodontal tissue regeneration by transplantation of bone marrow mesenchymal stem cells. J. Periodontol..

[55] Hasegawa N., Kawaguchi H., Hirachi A., Takeda K., Mizuno N., Nishimura M., Koike C., Tsuji K., Iba H., Kato Y. (2006). Behavior of transplanted bone marrow–derived mesenchymal stem cells in periodontal defects. J. Periodontol..

[56] Yamada Y., Ueda M., Hibi H., Baba S. (2006). A novel approach to periodontal tissue regeneration with mesenchymal stem cells and platelet-rich plasma using tissue engineering technology: A clinical case report. Int. J. Periodontics Restor. Dent..

[57] Lin N.H., Gronthos S., Mark Bartold P. (2009). Stem cells and future periodontal regeneration. Periodontol. 2000.

[58] Yoshida T., Washio K., Iwata T., Okano T., Ishikawa I. (2012). Current status and future development of cell transplantation therapy for periodontal tissue regeneration. Int. J. Dent..

[59] Tsumanuma Y., Iwata T., Washio K., Yoshida T., Yamada A., Takagi R., Ohno T., Lin K., Yamato M., Ishikawa I. (2011). Comparison of different tissue-derived stem cell sheets for periodontal regeneration in a canine 1-wall defect model. Biomaterials.

[60] Chen F.M., Sun H.H., Lu H., Yu Q. (2012). Stem cell-delivery therapeutics for periodontal tissue regeneration. Biomaterials.

[61] Yamada Y., Nakamura S., Ito K., Sugito T., Yoshimi R., Nagasaka T., Ueda M. (2010). A feasibility of useful cell-based therapy by bone regeneration with deciduous tooth stem cells, dental pulp stem cells, or bone-marrow-derived mesenchymal stem cells for clinical study using tissue engineering technology. Tissue Eng. Part A.

[62] Wada N., Menicanin D., Shi S., Bartold P.M., Gronthos S. (2009). Immunomodulatory properties of human periodontal ligament stem cells. J. Cell. Physiol..

[63] Ding G., Liu Y., Wang W., Wei F., Liu D., Fan Z., An Y., Zhang C., Wang S. (2010). Allogeneic periodontal ligament stem cell therapy for periodontitis in swine. Stem Cells.

[64] Nuñez J., Sanchez N., Vignoletti F., Sanz-Martin I., Caffesse R., Santamaria S., Garcia-Sanz J.A., Sanz M. (2018). Cell therapy with allogenic canine periodontal ligament-derived cells in periodontal regeneration of critical size defects. J. Clin. Periodontol..

[65] Garg T., Singh O., Arora S., Murthy R. (2012). Scaffold: A novel carrier for cell and drug delivery. Crit. Rev. Ther. Drug Carr. Syst..

[66] Amini A.R., Laurencin C.T., Nukavarapu S.P. (2012). Bone tissue engineering: Recent advances and challenges. Crit. Rev. Biomed. Eng..

[67] Hu L., Liu Y., Wang S. (2018). Stem cell-based tooth and periodontal regeneration. Oral Dis..

[68] Baik H.S., Park J., Lee K.J., Chung C. (2014). Local application of periodontal ligament stromal cells promotes soft tissue regeneration. Oral Dis..

[69] Hasegawa M., Yamato M., Kikuchi A., Okano T., Ishikawa I. (2005). Human periodontal ligament cell sheets can regenerate periodontal ligament tissue in an athymic rat model. Tissue Eng..

[70] Chen F.-M., Gao L.-N., Tian B.-M., Zhang X.-Y., Zhang Y.-J., Dong G.-Y., Lu H., Chu Q., Xu J., Yu Y. (2016). Treatment of periodontal intrabony defects using autologous periodontal ligament stem cells: A randomized clinical trial. Stem Cell Res. Ther..

[71] Kim S., Choi S.-I., Kim G.-H., Imm J.-Y. (2019). Anti-inflammatory effect of Ecklonia cava extract on Porphyromonas gingivalis lipopolysaccharide-stimulated macrophages and a periodontitis rat model. Nutrients.

[72] Magnussen S.N., Hadler-Olsen E., Costea D.E., Berg E., Jacobsen C.C., Mortensen B., Salo T., Martinez-Zubiaurre I., Winberg J.-O., Uhlin-Hansen L. (2017). Cleavage of the urokinase receptor (uPAR) on oral cancer cells: Regulation by transforming growth factor–β1 (TGF-β1) and potential effects on migration and invasion. BMC Cancer.

[73] Ehlers M.R. (2000). CR3: A general purpose adhesion-recognition receptor essential for innate immunity. Microbes Infect..

[74] Gaurilcikaite E., Renton T., Grant A. (2017). The paradox of painless periodontal disease. Oral Dis..

[75] Li Q., Valerio M.S., Kirkwood K.L. (2012). MAPK usage in periodontal disease progression. J. Signal Transduct..

[76] Wang L., Wu F., Song Y., Duan Y., Jin Z. (2018). Erythropoietin induces the osteogenesis of periodontal mesenchymal stem cells from healthy and periodontitis sources via activation of the p38 MAPK pathway. Int. J. Mol. Med..

[77] Derrigo M., Cestelli A., Savettieri G., Di Liegro I. (2000). RNA-protein interactions in the control of stability and localization of messenger RNA. Int. J. Mol. Med..

[78] Wang F., Guan M., Wei L., Yan H. (2019). IL-18 promotes the secretion of matrix metalloproteinases in human periodontal ligament fibroblasts by activating NF-κB signaling. Mol. Med. Rep..

[79] Zhao Q., Shepherd E.G., Manson M.E., Nelin L.D., Sorokin A., Liu Y. (2005). The role of mitogen-activated protein kinase phosphatase-1 in the response of alveolar macrophages to lipopolysaccharide: Attenuation of proinflammatory cytokine biosynthesis via feedback control of p38. J. Biol. Chem..

[80] Grzybowska E.A., Wilczynska A., Siedlecki J.A. (2001). Regulatory functions of 3’UTRs. Biochem. Biophys. Res. Commun..

[81] Lin J., Huang J., Zhang Z., Yu X.M., Cai X., Liu C. (2022). Periodontal ligament cells under mechanical force regulate local immune homeostasis by modulating Th17/Treg cell differentiation. Clin. Oral Investig..

[82] Darveau R.P., Pham T.-T.T., Lemley K., Reife R.A., Bainbridge B.W., Coats S.R., Howald W.N., Way S.S., Hajjar A.M. (2004). Porphyromonas gingivalis lipopolysaccharide contains multiple lipid A species that functionally interact with both toll-like receptors 2 and 4. Infect. Immun..

[83] Bonito A.J., Lux L., Lohr K.N. (2005). Impact of local adjuncts to scaling and root planing in periodontal disease therapy: A systematic review. J. Periodontol..

[84] Hajishengallis G., Ratti P., Harokopakis E. (2005). Peptide mapping of bacterial fimbrial epitopes interacting with pattern recognition receptors. J. Biol. Chem..

[85] Kirkwood K.L., Cirelli J.A., Rogers J.E., Giannobile W.V. (2007). Novel host response therapeutic approaches to treat periodontal diseases. Periodontology 2000.

[86] Isola G., Polizzi A., Patini R., Ferlito S., Alibrandi A., Palazzo G. (2020). Association among serum and salivary A. actinomycetemcomitans specific immunoglobulin antibodies and periodontitis. BMC Oral Health.

[87] Ambili R., Santhi W., Janam P., Nandakumar K., Pillai M.R. (2005). Expression of activated transcription factor nuclear factor-κB in periodontally diseased tissues. J. Periodontol..

[88] May M.J., Ghosh S. (1998). Signal transduction through NF-κB. Immunol. Today.

[89] Eapen D.J., Manocha P., Ghasemzadeh N., Patel R.S., Al Kassem H., Hammadah M., Veledar E., Le N.A., Pielak T., Thorball C.W. (2014). Soluble urokinase plasminogen activator receptor level is an independent predictor of the presence and severity of coronary artery disease and of future adverse events. J. Am. Heart Assoc..

[90] Parasrampuria D.A., de Boer P., Desai-Krieger D., Chow A.T., Jones C.R. (2003). Single-dose pharmacokinetics and pharmacodynamics of RWJ 67657, a specific p38 mitogen-activated protein kinase inhibitor: A first-in-human study. J. Clin. Pharmacol..

[91] Hoffmann E., Dittrich-Breiholz O., Holtmann H., Kracht M. (2002). Multiple control of interleukin-8 gene expression. J. Leukoc. Biol..

[92] Rakic M., Monje A., Radovanovic S., Petkovic-Curcin A., Vojvodic D., Tatic Z. (2020). Is the personalized approach the key to improve clinical diagnosis of peri-implant conditions? The role of bone markers. J. Periodontol..

[93] Neuenhahn M., Busch D.H. (2007). Unique functions of splenic CD8α+ dendritic cells during infection with intracellular pathogens. Immunol. Lett..

[94] Pavičič M., Van Winkelhoff A., Douqué N., Steures R., De Graaff J. (1994). Microbiological and clinical effects of metronidazole and amoxicillin in Actinobacillus actinomycetemcomitans associated periodontitis: A 2-year evaluation. J. Clin. Periodontol..

[95] Zhu C., Zhao Y., Wu X., Qiang C., Liu J., Shi J., Gou J., Pei D., Li A. (2020). The therapeutic role of baicalein in combating experimental periodontitis with diabetes via Nrf2 antioxidant signaling pathway. J. Periodontal Res..

[96] Kumar G., Roger P.-M. (2019). From crosstalk between immune and bone cells to bone erosion in infection. Int. J. Mol. Sci..

[97] Preshaw P.M. (2018). Host modulation therapy with anti-inflammatory agents. Periodontol. 2000.

[98] Culshaw S., McInnes I.B., Liew F.Y. (2011). What can the periodontal community learn from the pathophysiology of rheumatoid arthritis?. J. Clin. Periodontol..

[99] Mayer Y., Balbir-Gurman A., Machtei E.E. (2009). Anti-tumor necrosis factor-alpha therapy and periodontal parameters in patients with rheumatoid arthritis. J. Periodontol..

[100] Mayer Y., Elimelech R., Balbir-Gurman A., Braun-Moscovici Y., Machtei E.E. (2013). Periodontal condition of patients with autoimmune diseases and the effect of anti-tumor necrosis factor-α therapy. J. Periodontol..

[101] Golub L.M., Lee H.M. (2020). Periodontal therapeutics: Current host-modulation agents and future directions. Periodontology 2000.

[102] Nakashima T., Kobayashi Y., Yamasaki S., Kawakami A., Eguchi K., Sasaki H., Sakai H. (2000). Protein expression and functional difference of membrane-bound and soluble receptor activator of NF-κB ligand: Modulation of the expression by osteotropic factors and cytokines. Biochem. Biophys. Res. Commun..

[103] Hienz S.A., Paliwal S., Ivanovski S. (2015). Mechanisms of bone resorption in periodontitis. J. Immunol. Res..

[104] Bi C.S., Sun L.J., Qu H.L., Chen F., Tian B.M., Chen F.M. (2019). The relationship between T-helper cell polarization and the RANKL/OPG ratio in gingival tissues from chronic periodontitis patients. Clin. Exp. Dent. Res..

[105] Currò M., Matarese G., Isola G., Caccamo D., Ventura V., Cornelius C., Lentini M., Cordasco G., Ientile R. (2014). Differential expression of transglutaminase genes in patients with chronic periodontitis. Oral Dis..

[106] Ansari Moghadam S., Sarani S., Alijani E., Ansari Moghadam A. (2019). The effect of Phase 1 periodontal treatment on the salivary RANKL/OPG ratio in severe chronic periodontitis. Clin. Cosmet. Investig. Dent..

[107] Sattari M., Taheri R.A., ArefNezhad R., Motedayyen H. (2021). The expression levels of MicroRNA-146a, RANKL and OPG after non-surgical periodontal treatment. BMC Oral Health.

[108] Belibasakis G.N., Bostanci N. (2012). The RANKL-OPG system in clinical periodontology. J. Clin. Periodontol..

[109] Tsukasaki M. (2021). RANKL and osteoimmunology in periodontitis. J. Bone Miner. Metab..

[110] Nagarajan R., Miller C.S., Dawson D., Al-Sabbagh M., Ebersole J.L. (2015). Patient-Specific Variations in Biomarkers across Gingivitis and Periodontitis. PLoS ONE.

[111] Bostanci N., Ilgenli T., Emingil G., Afacan B., Han B., Töz H., Berdeli A., Atilla G., McKay I.J., Hughes F.J. (2007). Differential expression of receptor activator of nuclear factor-kappaB ligand and osteoprotegerin mRNA in periodontal diseases. J Periodontal. Res..

[112] Wara-aswapati N., Surarit R., Chayasadom A., Boch J.A., Pitiphat W. (2007). RANKL upregulation associated with periodontitis and Porphyromonas gingivalis. J. Periodontol..

[113] Reddi D., Bostanci N., Hashim A., Aduse-Opoku J., Curtis M.A., Hughes F.J., Belibasakis G.N. (2008). Porphyromonas gingivalis regulates the RANKL-OPG system in bone marrow stromal cells. Microbes Infect..

[114] Gao W., Tang H., Wang D., Zhou X., Song Y., Wang Z. (2020). Effect of short-term vitamin D supplementation after nonsurgical periodontal treatment: A randomized, double-masked, placebo-controlled clinical trial. J. Periodontal Res..

[115] Perayil J., Menon K.S., Kurup S., Thomas A.E., Fenol A., Vyloppillil R., Bhaskar A., Megha S. (2015). Influence of vitamin D & calcium supplementation in the management of periodontitis. J. Clin. Diagn. Res..

[116] Hammarström L. (1997). Enamel matrix, cementum development and regeneration. J. Clin. Periodontol..

[117] Miguel M.M.V., Mathias-Santamaria I.F., Rossato A., Ferraz L.F.F., Rangel T.P., Casarin R.C.V., Tatakis D.N., Santamaria M.P. (2021). Enamel matrix derivative effects on palatal mucosa wound healing: Randomized clinical trial. J. Periodontal Res..

[118] Miron R., Dard M., Weinreb M. (2015). Enamel matrix derivative, inflammation and soft tissue wound healing. J. Periodontal Res..

[119] Heijl L., Heden G., Svardstrom G., Ostgren A. (1997). Enamel matrix derivative (EMDOGAIN) in the treatment of intrabony periodontal defects. J. Clin. Periodontol..

[120] Venezia E., Goldstein M., Boyan B., Schwartz Z. (2004). The use of enamel matrix derivative in the treatment of periodontal defects: A literature review and meta-analysis. Crit. Rev. Oral Biol. Med..

[121] Esposito M., Coulthard P., Thomsen P., Worthington H.V. (2004). Enamel matrix derivative for periodontal tissue regeneration in treatment of intrabony defects: A Cochrane systematic review. J. Dent. Educ..

[122] Bommer C., Waller T., Hilbe M., Wiedemeier D., Meyer N., Mathes S., Jung R. (2022). Efficacy and safety of P11-4 for the treatment of periodontal defects in dogs. Clin. Oral Investig..

[123] Rojas M.A., Marini L., Pilloni A., Sahrmann P. (2019). Early wound healing outcomes after regenerative periodontal surgery with enamel matrix derivatives or guided tissue regeneration: A systematic review. BMC Oral Health.

[124] Sculean A., Windisch P., Döri F., Keglevich T., Molnár B., Gera I. (2007). Emdogain in regenerative periodontal therapy. A review of the literature. Fogorv. Szle..

[125] Christgau M., Bader N., Felden A., Gradl J., Wenzel A., Schmalz G. (2002). Guided tissue regeneration in intrabony defects using an experimental bioresorbable polydioxanon (PDS) membrane: A 24-month split-mouth study. J. Clin. Periodontol..

[126] Szatmári P., Gera I. (2014). Treatment of localized intrabony periodontal defects with enamel matrix derivative (Emdogain). Case series. Fogorv. Szle..

[127] Vasil’ev Y.L., Kytko O., Smetneva N., Goloborodova I., Nelipa M. (2019). The morphofunctional features of platelets against the background of metabolic syndrome in patients with generalized marginal periodontitis. Diabetes Metab. Syndr. Clin. Res. Rev..

[128] Mohan S.P., Jaishangar N., Devy S., Narayanan A., Cherian D., Madhavan S.S. (2019). Platelet-Rich Plasma and Platelet-Rich Fibrin in Periodontal Regeneration: A Review. J. Pharm. Bioallied. Sci..

[129] Döri F., Kovács V., Arweiler N.B., Huszár T., Gera I., Nikolidakis D., Sculean A. (2009). Effect of platelet-rich plasma on the healing of intrabony defects treated with an anorganic bovine bone mineral: A pilot study. J. Periodontol..

[130] Duarte W.R., Iimura T., Takenaga K., Ohya K., Ishikawa I., Kasugai S. (1999). Extracellular role of S100A4 calcium-binding protein in the periodontal ligament. Biochem. Biophys. Res. Commun..

[131] Gupta S.J., Jhingran R., Gupta V., Bains V.K., Madan R., Rizvi I. (2014). Efficacy of platelet-rich fibrin vs. enamel matrix derivative in the treatment of periodontal intrabony defects: A clinical and cone beam computed tomography study. J. Int. Acad. Periodontol..

[132] Miron R.J., Moraschini V., Fujioka-Kobayashi M., Zhang Y., Kawase T., Cosgarea R., Jepsen S., Bishara M., Canullo L., Shirakata Y. (2021). Use of platelet-rich fibrin for the treatment of periodontal intrabony defects: A systematic review and meta-analysis. Clin. Oral Investig..

[133] Bürgers R., Gerlach T., Hahnel S., Schwarz F., Handel G., Gosau M. (2010). In vivo and in vitro biofilm formation on two different titanium implant surfaces. Clin. Oral Implant. Res..

[134] Lynch S.E., Buser D., Hernandez R.A., Weber H.P., Stich H., Fox C.H., Williams R.C. (1991). Effects of the platelet-derived growth factor/insulin-like growth factor-I combination on bone regeneration around titanium dental implants. Results of a pilot study in beagle dogs. J. Periodontol..

[135] Rutherford R., Niekrash C., Kennedy J., Charette M. (1992). Platelet-derived and insulin-like growth factors stimulate regeneration of periodontal attachment in monkeys. J. Periodontal Res..

[136] Kusuyama J., Nakamura T., Ohnishi T., Albertson B.G., Ebe Y., Eiraku N., Noguchi K., Matsuguchi T. (2019). Low-intensity pulsed ultrasound promotes bone morphogenic protein 9-induced osteogenesis and suppresses inhibitory effects of inflammatory cytokines on cellular responses via Rho-associated kinase 1 in human periodontal ligament fibroblasts. J. Cell. Biochem..

[137] Barboza E.P., Caúla A.L., Caúla F.d.O., de Souza R.O., Neto L.G., Sorensen R.G., Li X.J., Wikesjö U.M. (2004). Effect of recombinant human bone morphogenetic protein-2 in an absorbable collagen sponge with space-providing biomaterials on the augmentation of chronic alveolar ridge defects. J. Periodontol..

[138] Wang B., Mastrogiacomo S., Yang F., Shao J., Ong M.M.A., Chanchareonsook N., Jansen J.A., Walboomers X.F., Yu N. (2019). Application of BMP-Bone cement and FGF-Gel on periodontal tissue regeneration in nonhuman primates. Tissue Eng. Part C Methods.

[139] Zang S., Mu R., Chen F., Wei X., Zhu L., Han B., Yu H., Bi B., Chen B., Wang Q. (2019). Injectable chitosan/β-glycerophosphate hydrogels with sustained release of BMP-7 and ornidazole in periodontal wound healing of class III furcation defects. Mater. Sci. Eng. C.

[140] Gao Y., Zhang M., Tian X., Wang M., Zhang F. (2019). Experimental animal study on BMP-3 expression in periodontal tissues in the process of orthodontic tooth movement. Exp. Ther. Med..

[141] Kang W., Liang Q., Du L., Shang L., Wang T., Ge S. (2019). Sequential application of bFGF and BMP-2 facilitates osteogenic differentiation of human periodontal ligament stem cells. J. Periodontal Res..

[142] Kanaya S., Nemoto E., Ebe Y., Somerman M.J., Shimauchi H. (2010). Elevated extracellular calcium increases fibroblast growth factor-2 gene and protein expression levels via a cAMP/PKA dependent pathway in cementoblasts. Bone.

[143] Bermúdez M., Imaz-Rosshandler I., Rangel-Escareño C., Zeichner-David M., Arzate H., Mercado-Celis G.E. (2015). CEMP1 Induces Transformation in Human Gingival Fibroblasts. PLoS ONE.

[144] Lim H.C., Cha B.Y., Song S.U., Yun J.H. (2018). Harmine promotes periodontal ligament cell-induced tissue regeneration. Oral Dis..

[145] Carmona-Rodríguez B., Álvarez-Pérez M.A., Narayanan A.S., Zeichner-David M., Reyes-Gasga J., Molina-Guarneros J., García-Hernández A.L., Suárez-Franco J.L., Chavarría I.G., Villarreal-Ramírez E. (2007). Human Cementum Protein 1 induces expression of bone and cementum proteins by human gingival fibroblasts. Biochem. Biophys. Res. Commun..

[146] Mada Y., Miyauchi M., Oka H., Kitagawa M., Sakamoto K., Iizuka S., Sato S., Noguchi K., Somerman M., Takata T. (2006). Effects of endogenous and exogenous prostaglandin E2 on the proliferation and differentiation of a mouse cementoblast cell line (OCCM-30). J. Periodontol..

[147] Pham L., Bezouglaia O., Camargo P., Nervina J., Tetradis S. (2007). Prostanoids induce egr1 gene expression in cementoblastic OCCM cells. J. Periodontal Res..

[148] Kajiya M., Ichimonji I., Min C., Zhu T., Jin J.O., Yu Q., Almazrooa S.A., Cha S., Kawai T. (2012). Muscarinic type 3 receptor induces cytoprotective signaling in salivary gland cells through epidermal growth factor receptor transactivation. Mol. Pharm..

[149] Kajiya M., Shiba H., Fujita T., Takeda K., Uchida Y., Kawaguchi H., Kitagawa M., Takata T., Kurihara H. (2009). Brain-derived neurotrophic factor protects cementoblasts from serum starvation-induced cell death. J. Cell. Physiol..

[150] Kajiya M., Shiba H., Fujita T., Ouhara K., Takeda K., Mizuno N., Kawaguchi H., Kitagawa M., Takata T., Tsuji K. (2008). Brain-derived neurotrophic factor stimulates bone/cementum-related protein gene expression in cementoblasts. J. Biol. Chem..

[151] Offenbacher S., Barros S.P., Beck J.D. (2008). Rethinking periodontal inflammation. J. Periodontol..

[152] Asa’ad F., Bollati V., Pagni G., Castilho R.M., Rossi E., Pomingi F., Tarantini L., Consonni D., Giannobile W.V., Rasperini G. (2017). Evaluation of DNA methylation of inflammatory genes following treatment of chronic periodontitis: A pilot case–control study. J. Clin. Periodontol..

[153] Oliveira N.F., Damm G.R., Andia D.C., Salmon C., Nociti Jr F.H., Line S.R., De Souza A.P. (2009). DNA methylation status of the IL8 gene promoter in oral cells of smokers and non-smokers with chronic periodontitis. J. Clin. Periodontol..

[154] Carty E., Nickols C., Feakins R., Rampton D. (2002). Thromboxane synthase immunohistochemistry in inflammatory bowel disease. J. Clin. Pathol..

[155] Liu C., Mo L., Niu Y., Li X., Zhou X., Xu X. (2017). The role of reactive oxygen species and autophagy in periodontitis and their potential linkage. Front. Physiol..

[156] Hajishengallis G., Chavakis T., Lambris J.D. (2020). Current understanding of periodontal disease pathogenesis and targets for host-modulation therapy. Periodontol. 2000.

[157] Kim H.-N., Ha H., Lee J.-H., Jung K., Yang D., Woo K.M., Lee Z.H. (2009). Trichostatin A inhibits osteoclastogenesis and bone resorption by suppressing the induction of c-Fos by RANKL. Eur. J. Pharmacol..

[158] Takada Y., Gillenwater A., Ichikawa H., Aggarwal B.B. (2006). Suberoylanilide hydroxamic acid potentiates apoptosis, inhibits invasion, and abolishes osteoclastogenesis by suppressing nuclear factor-κB activation. J. Biol. Chem..

[159] Cantley M., Fairlie D., Bartold P., Rainsford K., Le G., Lucke A., Holding C., Haynes D. (2011). Inhibitors of histone deacetylases in class I and class II suppress human osteoclasts in vitro. J. Cell. Physiol..

[160] Offenbacher S., Divaris K., Barros S.P., Moss K.L., Marchesan J.T., Morelli T., Zhang S., Kim S., Sun L., Beck J.D. (2016). Genome-wide association study of biologically informed periodontal complex traits offers novel insights into the genetic basis of periodontal disease. Hum. Mol. Genet..

[161] Jurdziński K.T., Potempa J., Grabiec A.M. (2020). Epigenetic regulation of inflammation in periodontitis: Cellular mechanisms and therapeutic potential. Clin. Epigenetics.

